# Formation of Diamane Nanostructures in Bilayer Graphene on Langasite under Irradiation with a Focused Electron Beam

**DOI:** 10.3390/nano12244408

**Published:** 2022-12-10

**Authors:** Eugenii V. Emelin, Hak Dong Cho, Vitaly I. Korepanov, Liubov A. Varlamova, Sergey V. Erohin, Deuk Young Kim, Pavel B. Sorokin, Gennady N. Panin

**Affiliations:** 1 Institute of Microelectronics Technology and High-Purity Materials, Russian Academy of Sciences, Chernogolovka, 142432 Moscow, Russia; 2 Quantum-Functional Semiconductor Research Center, Dongguk University, Seoul 04620, Republic of Korea; 3 Laboratory of Digital Material Science, National University of Science and Technology MISIS, 119049 Moscow, Russia; 4 Department of Semiconductors and Dielectrics, National University of Science and Technology MISIS, 119049 Moscow, Russia; 5 Division of Physics and Semiconductor Science, Dongguk University, Seoul 04620, Republic of Korea

**Keywords:** graphene, diamane, bias-enhanced nucleation, chemically induced phase transition

## Abstract

In the presented paper, we studied bilayer CVD graphene transferred to a langasite substrate and irradiated with a focused electron beam through a layer of polymethyl methacrylate (PMMA). Changes in the Raman spectra and an increase in the electrical resistance of bigraphene after irradiation indicate a local phase transition associated with graphene diamondization. The results are explained in the framework of the theory of a chemically induced phase transition of bilayer graphene to diamane, which can be associated with the release of hydrogen and oxygen atoms from PMMA and langasite due to the “knock-on” effect, respectively, upon irradiation of the structure with an electron beam. Theoretical calculations of the modified structure of bigraphene on langasite and the experimental evaluation of sp^3^-hybridized carbon fraction indicate the formation of diamane nanoclusters in the bigraphene irradiated regions. This result can be considered as the first realization of local tunable bilayer graphene diamondization.

## 1. Introduction

Graphene, due to its outstanding properties, has the widest range of applications (including electronics, optics [1], photovoltaics [2], spintronics [3], etc.). One of its fascinating fields of application is its use as a basis for the creation of new 2D materials. For example, the connection of multilayered graphene layers leads to the formation of the thinnest diamond film, diamane [4]. Such a way of obtaining diamane seems to be the most promising. Indeed, various methods of diamond production have been developed, among which HPHT and CVD are the most widely used ones. However, when the size is reduced, surface effects come into play and dramatically affect the diamond stability. Thus, new synthesis techniques are required.

Indeed, the theory suggests that reducing the thickness of the diamond film to several layers leads to instability of the structure and its decomposition into multilayered graphene [5]. This prediction is well supported by experiments [6,7,8] where the direct pressure in diamond anvil cells was used to induce conversion of the whole graphene flake. The obtained value of phase transition pressure was much higher than in the bulk case, which reflects the increase in the film surface energy. Moreover, no diamondization has occurred in the case of bilayered films in full agreement with the theoretical predictions [8,9].

On the other hand, the prevailing surface contribution allows the modification of the graphene structure by surface functionalization, e.g., by H [4,5,10,11], OH [11,12], and F [11,13] which leads to the connection of the layers to form diamane [4]. The effect of 2D diamond formation by purely chemical routes was called a chemically induced phase transition [5]. The developed theory was extensively verified experimentally [14] (most often by the hydrogen [15], fluorine [16,17,18], or water [9,19,20] treatment of graphene). Adsorption of reference atoms regulated by external conditions, by choosing appropriate temperatures and pressures, changes the structure of the film globally. However, this approach is not suitable when local manipulation of atomic geometry is required. It can be realized by using the biased enhanced nucleation (BEN) approach [21] allowing phase transformation by exposing direct electron irradiation of selected regions. According to the reference data, it can be an effective technique for restructuring various two-dimensional [22] nanomaterials.

The use of electron irradiation for realizing the chemically induced phase transition was demonstrated in Ref. [23], where the induced formation of diamond nanocluster in the carbon network was shown. Hydrogen atoms displaced from the dodecyl groups by the “knock-on” effect penetrate the layered carbon and form a dense amorphous hydrogenated carbon (C:H) phase with the final precipitation of sp*^3^* carbon clusters. It is attractive to extend this approach to the two-dimensional case, where an easily accessible surface allows local phase-state tuning and enables the formation of a heterostructure consisting of regions with different conductivities. The latter can become the basis for nanoscale electronic devices.

In this study, we have considered the chemically induced phase transition induced by the “knock-on” effect in bilayer graphene sandwiched in langasite and PMMA. In the first part of the paper, we describe the synthesis conditions and show how electron irradiation affects the structure under study depending on the parameters of the electron beam. Next, we investigate transport properties by measuring the I–V characteristics of the nanostructure and show that the resistance of the graphene bilayer after irradiation with an electron beam increases significantly. Then, we study the structural characteristics of the material by estimating the content of sp*^3^* carbon in irradiated bigraphene and show the formation of diamondized regions responsible for the resistance increase. Finally, we present a theoretical model and show that its structural and electronic properties agree well with the experiment.

## 2. Methods

### 2.1. Experimental Methods

Graphene monolayers were obtained by chemical vapor deposition (CVD) on a copper catalyst foil. The CVD reactor we used was a horizontal quartz tube 2400 mm long and 152 mm in diameter. This quartz tube was placed in a six-zone furnace 1500 mm long. A 25 µm thick copper foil (from Alfa Aesar, 99.999%, 10 × 30 cm^2^) was loaded into a CVD reactor and evacuated to base vacuum pressure (<10^−4^ Torr). The temperature in the chamber with a mixture of Ar (2000 cm^3^/min) and H_2_ (30 cm^3^/min) was raised to 1060 °C at an operating pressure of 470 Torr. The copper foil was annealed at 1060 °C with a mixture of Ar (2000 cm^3^/min) and H_2_ (30 cm^3^/min) at an operating pressure of 470 Torr for two hours. Graphene layers began to grow on copper foil at 1020 °C when a mixture of methane and hydrogen (CH_4_ = 40 cm^3^/min and H_2_ = 100 cm^3^/min) and 2000 cm^3^/min Ar as the carrier gas was introduced into the reaction chamber at 600 mTorr. After 30 min, the reaction chamber was cooled to room temperature at an average rate of 14 °C/min at the same flow rates of Ar/H_2_ gas, without methane.

Two grown graphene monolayers were transferred to a polished surface of a lanthanum gallium silicate (La_3_Ga_5_SiO_14_) (langasite) substrate using a 2 µm thick electron-resist polymethyl methacrylate (950 K C4; Microchem), which was spin-coated onto the synthesized graphene at 3000 rpm for 100 s. PMMA was dried in an oven at 120 °C for 10 min. Since graphene layers grew on both sides of the copper foil during chemical vapor deposition, the removal of graphene layers from the reverse side of the foil was carried out in oxygen plasma with a power of 60 W for 10 min. Then the copper foil was removed in a copper etchant (CE-100, Transene) for 40–60 min. To wash graphene after copper etching, PMMA/graphene was placed successively in two water baths for 20 min at a time. After washing with distilled water, the PMMA/graphene layer was transferred onto an LGS substrate. The PMMA was then removed with acetone in an ultrasonic bath for 20 min, leaving single layer graphene (SLG) on the LGS substrate. Then, the SLG on LGS was washed with a 30% HCl solution at 60 °C for 30 min to remove residual Fe^3+^ ions. As a result, a pure high-quality graphene layer with a low defect density was prepared on the LGS substrate. The next layer of graphene was transferred using the same procedure. Before transferring the upper layer, the surface of the lower layer was thoroughly cleaned with solvents and an ultrasonic bath to ensure a tight fit of the graphene monolayers to each other.

Graphene layers on the substrates were characterized by Raman scattering using a CRM 200 spectrometer (WiTec, Germany) with a 100× objective (Olympus, NA 0.9), a 532 nm (488 nm) laser with a power of 1 mW (2.5 mW, 50 mW), and a GX polarized filter-AN360 (Olympus). Each spectrum was obtained by 10 measurements within 10 s of the accumulation time. For each sample, 3 to 6 analyzes were performed at different locations. Then, a layer of PMMA-950 resist, 300 nm thick, was deposited on the LGS substrate with bigraphene by centrifugation. These samples were irradiated using an EVO-50 scanning electron microscope equipped with Nanomaker electron beam control. The Raman spectra of nanostructures obtained by irradiation with a focused electron beam were measured using a Bruker Senterra micro-Raman system. The excitation wavelength was 532 nm; laser power at the sample point was 10 mW; the capture was 2 × 20 s at each point on the map. Transport measurements of nanostructures were carried out using a microprobe station EPS150TRIAX and a Keithley 2636B System SourceMeter^®^ SMU Instrument.

### 2.2. Computational Details

All calculations were performed within density functional theory (DFT) [24,25] in the generalized gradient approximation via Perdew–Burke–Ernzerhof (PBE) parametrization [26] as implemented in the VASP package [27,28,29]. The plane–wave cutoff energy was set to 400 eV, while the Brillouin zone was sampled using an 2 × 1 × 1 Monkhorst–Pack grid [30]. For the density of electronic states (DOS) calculation we used 4 × 2 × 1 the Brillouin zone sampling. Atomic structure relaxation was carried out until the maximum interatomic force became less than 0.02 eV/Å. To avoid interaction between the neighboring images of the studied slab, the translation vector along *c* axis was set to be greater than 15 Å. The langasite model has a thickness of ~1 nm (two unit cells) with a frozen bottom layer.

## 3. Results and Discussion

**Figure 1:**
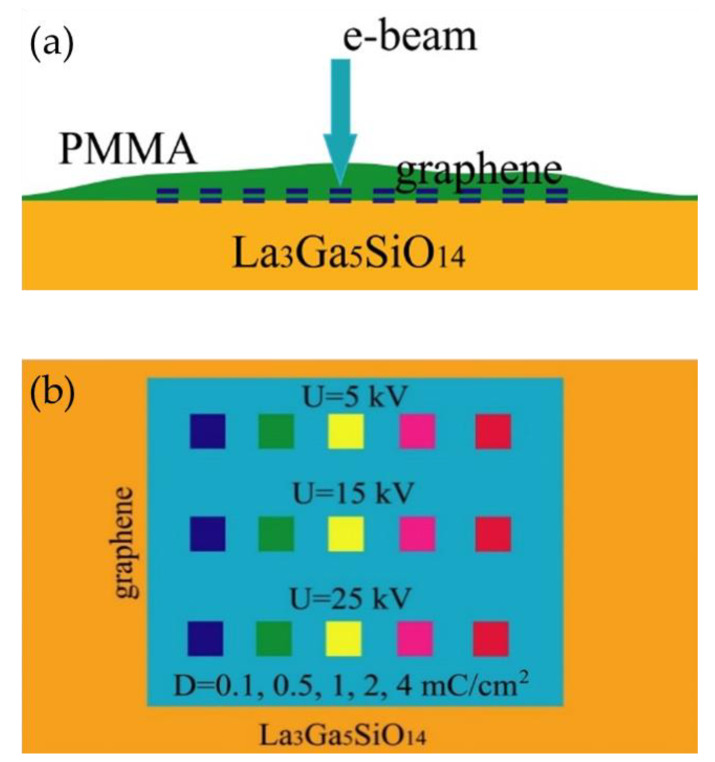
Schematic representation of a structure consisting of two PMMA-coated graphene layers on an LGS substrate (**a**) and scheme of its irradiation with an electron beam (**b**). Graphene layers are separated in (**a**) for clarity.

A schematic representation of the irradiation experiment is shown in Figure 1. Two monolayers of graphene were transferred onto the LGS substrate (see Figure 1a). The LGS crystal was chosen as a substrate for bigraphene. As was previously found, when the LGS surface is treated at a sufficiently high temperature or irradiated with a focused electron beam, the exposed area of the crystal surface loses oxygen. Using photolithography, a photoresist mask with an area of 1 mm^2^ was fabricated. Unmasked graphene was removed from the substrate in oxygen plasma. Thus, a double layer of graphene with an area of 1 mm^2^ remained on the LGS substrate, to which four contacts, 100 nm thick, consisting of an alloy of gold and chromium in a ratio of 20:1, were attached using electron beam lithography (see Figure 1b). At the final stage of structure formation, a layer of PMMA-950 resist 300 nm thick was deposited on the sample surface and irradiated with a focused electron beam at various beam parameters. It is well known that the PMMA molecule is destroyed by the action of an electron beam with the release of hydrogen. PMMA at low doses of irradiation exhibits the properties of a positive resist, so the modification of graphene can be carried out only in the region of electron beam irradiation. It is preferable to the negative HSQ (H-SiO_x_) resist which was previously demonstrated as a source of hydrogen released during the development of the resist and forming sp*^3^* bonds with graphene [31,32]. The use of negative resist, especially H-SiO_x_, causes the problem of cleaning the treated layer from residual resist contamination. The use of PMMA solves the problem of graphene contamination as PMMA dissolves in acetone, almost without residue.

Bigraphene squares with an area of 0.25 mm^2^ were irradiated at three accelerating electron beam voltages of 5 kV, 15 kV, and 25 kV at a beam current I = 1 nA (Figure 1b). The irradiation dose D varied in the range from 0.1 mC/cm^2^ (the experimentally determined irradiation dose sufficient for the complete destruction of PMMA at U = 25 kV) to 4 mC/cm^2^. After irradiation, the irradiated areas were developed and examined. The analysis showed that, with an increase in the density of the implanted charge (irradiation dose), structural changes occurred in the double graphene layer. The use of doses above 1 mC/cm^2^ resulted in PMMA-950 crosslinking, so they were not used in subsequent experiments. With a decrease in the accelerating voltage, the threshold dose sufficient for resist crosslinking decreases due to an increase in the effective absorbed-beam energy. Therefore, the accelerating voltage of U = 25 kV was chosen as the optimal value.

**Figure 2:**
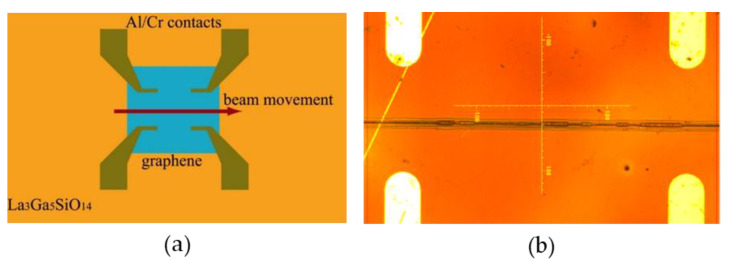
Scheme of the experimental sample (**a**) and optical image of the investigated LGS/bigraphene/Cr/Al/PMMA structure irradiated with an electron beam (U = 25 kV, D = 1 mC/cm^2^) along the line (~200 nm wide) between the Al/Cr electrodes (**b**).

For U = 25 kV, the maximum possible radiation dose that does not lead to crosslinking of the resist is 1 mC/cm^2^. Based on the data obtained, we chose the optimal conditions for a local chemically induced phase transition in bilayer graphene to form the bigraphene/diamane/bigraphene nanostructure and measured its transport characteristics. The scheme of the experimental sample and the image of the investigated LGS/bigraphene/Cr/Al/PMMA structure irradiated with an electron beam are shown in Figure 2.

As shown in Figure 2a, the irradiation line crossed the entire area of the graphene bilayer. Thus, in the case of a phase transition of bigraphene to diamane, as a result of the irradiation of bigraphene with an electron beam, a barrier for the transfer of charge carriers should form in the bigraphene/diamane/bigraphene nanostructure, which can be detected by transport measurements.

**Figure 3:**
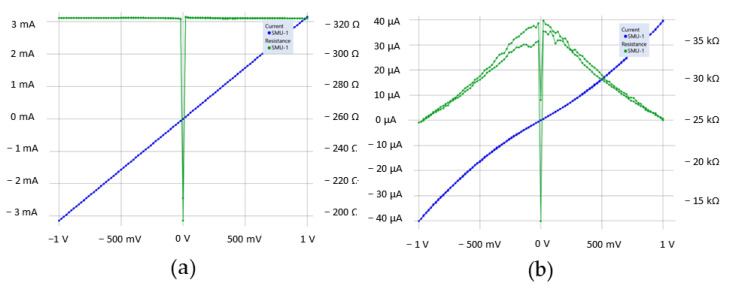
I–V (blue) and resistance change (green) in Al(Cr)/bigraphene/Al(Cr) structure unmodified (**a**) and modified by local electron beam irradiation (**b**) measured between the top and bottom electrodes, respectively, as shown schematically in Figure 2a.

Measurements of the I–V characteristics of the nanostructure between contacts to the graphene bilayer in the longitudinal direction to the irradiation line (Figure 3a) show a linear behavior (blue line) with resistance of 320 Ω (green line). Measurements of the I–V characteristics in the transverse direction of the irradiation line revealed the formation of a modified structure with a nonlinear character of charge carrier transport and an increase in resistance up to 38 kΩ (Figure 3b, blue and green lines, respectively).

Transport measurements show that the resistance of the graphene bilayer after irradiation with an electron beam increases significantly. The linear dependence of current on voltage in the bias voltage range from −1 to 1 V changes to a nonlinear one. This indicates the formation of a semiconductor phase with higher electrical resistance.

**Figure 4:**
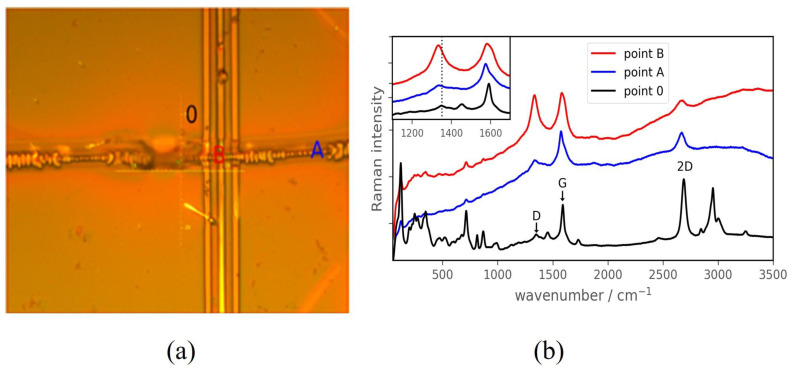
Optical image of the modified bigraphene regions irradiated with an electron beam (cross-like features) (**a**) and Raman spectra obtained at points 0, A, and B of a bigraphene/modified bigraphene/bigraphene structure made from PMMA/bigraphene on an LGS substrate treated with an electron beam at doses of 0, 0.5, and 1 mC/cm^2^, respectively (**b**). The inset in (**b**) shows the region of the D and G peaks with vertical line at 1352 cm^−1^ (position of the D-peak for point 0). Most Raman bands (unlabeled) belong to the LGS substrate. Intensity scaled for clarity (hence, the LGS bands have different intensities).

**Figure 5:**
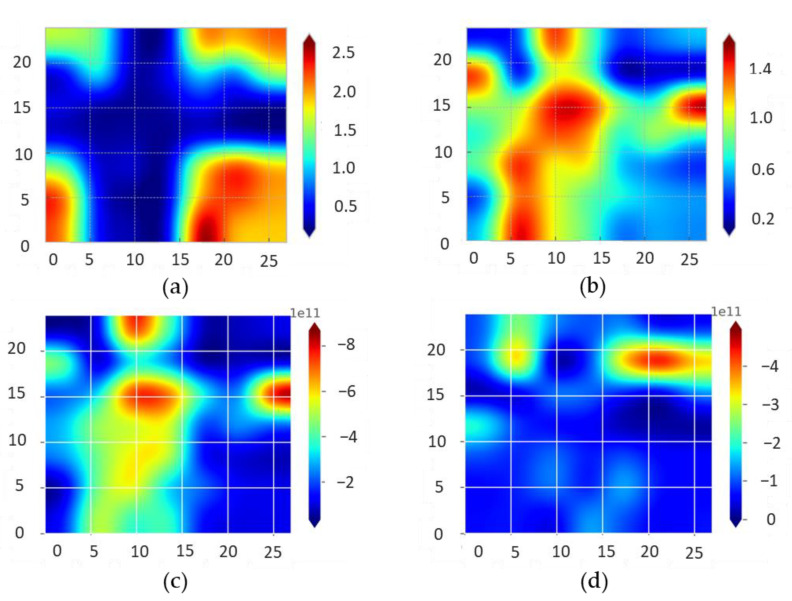
Intensity ratio maps of the 2D/G (**a**) and D/G (**b**) Raman bands of bilayer graphene after local electron irradiation (intersecting vertical and horizontal bands). sp^3^ defect density distribution (cm^−2^) estimated from D (**c**) and D′ (**d**) peaks.

To study the structural properties of bigraphene modified locally by irradiation with a focused electron beam, we used Raman spectroscopy. Raman mapping was conducted in the ~27 × 24 µm area around the cross shown in Figure 4a. The deconvolution of the graphene peaks was carried out after the baseline subtraction with the algorithm described in Ref. [33]. Raman modes are significantly modified in the irradiated regions of graphene. Among the most pronounced changes, the D peaks become higher while the intensity of the 2D peak decreases (Figure 4b and Figure 5a). The Raman spectrum in the non-irradiated region (0 point) clearly shows the main G and 2D modes for bigraphene. Irradiation with an electron beam leads to a clear decrease in the intensity of the 2D peak, and a pronounced increase in the D peak intensity (which is almost negligible in the 0 point). This indicates an increase in the sp*^3^*-hybridized carbon density. The width (FWHM) of the 2D band also shows a significant increase from 43 cm^−1^ for the initial bigraphene to ~100 cm^−1^ for the irradiated points. The Raman mode of carbon at ~1345 cm^−1^ is associated with defects responsible for the formation of sp*^3^*-hybridized regions, which is commonly observed in graphene oxide (GO). Previously, we have shown that electron beam irradiation of GO leads to its effective reduction with a decrease in the sp*^3^*-hybridized carbon density and a relative decrease in the intensity of the D peak without its shift [34]. In this work, we demonstrate that irradiation of a specially designed structure with an electron beam leads to a shift of the D-peak to 1335 cm^−1^ and an increase in intensity (Figure 4b, inset). We expect that for diamanes, the Raman peak should be at lower wavenumbers than the D-peak of graphene. A sign of diamond-like hybridization in few-layer graphene was observed as a peak at 1319–1337 cm^−1^ [35], while the graphene D-peak is located at ~1350 cm^−1^ [36]. Thus, the sp^3^-hybridized carbon concentration increases, and the characteristic peak shifts towards the diamane Raman mode.

To estimate the content of sp^3^ carbon in bigraphene irradiated with a locally focused electron beam, we used the approach from [37,38] where the dependence of the peaks D′ and D on the concentration and types of defects was studied. The following equation was proposed and its parameters were estimated for vacancies and sp^3^ defects [38]:

(1) $$\;n_{D}\left(cm^{-2}\right)=\frac{10^{14}}{\pi^{2}(C_{A}\left(r_{A}^{2}-r_{S}^{2}\right)+C_{S}r_{S}^{2}}\frac{I\left(x\right)}{I\left(G\right)},$$

For the D peak, *C_A_* = 4.2, *C_S_* = 0, *r_A_* = 3 nm, *r_S_* = 1 nm and for the D′ peak, *C_A_* = 0.5, *C_S_* = 0.33, *r_A_* = 2.6 nm, *r_S_* = 1.4 nm.

The *C_A_* parameter is a measure of the maximum possible value of the *I*(D)/*I*(G) ratio in graphene; the *C_S_* parameter is the value of the *I*(D)/*I*(G) ratio in the highly disordered limit; *r_S_* and *r_A_* are the radii of two circular areas measured from the defect site. The first length, *r_S_*, is the radius of the structurally disordered area around the defect, so it is expected to change from defect to defect. For distances larger than *r_S_* but shorter than *r_A_*, the lattice structure is preserved. However, the proximity to a defect causes the mixing of Bloch states near the **K** and **K**′ valleys of the graphene Brillouin zone and, therefore, breaking of selection rules and leading to an enhancement of the D band. If we accept that defects of the “vacancy” type can be neglected, then the concentration of sp^3^ carbon can be estimated from the ratio of the intensities *I*(D)/*I*(G) and *I*(D′)/*I*(G), which gives the maps shown in Figure 5b–d.

Our estimates show that the fraction of sp^3^ carbon in the irradiated region is about 10^12^ cm^–2^ (Figure 5c,d). When a certain local area of the sample is irradiated with a focused electron beam, hydrogen is released from the destroyed polymer on the one side, and oxygen from the LGS substrate on the other side. We suppose that active O and H atoms easily bind to the graphene surface, which leads to the corrugation of the layer with displacing of the neighboring atom from the plane owing to sp^3^ hybridization. Such behavior of graphene is typical upon the attachment of reference atoms to it. In the case of a single layer of graphene in this way, it is possible to obtain the “ultimate diamond slab” [39] (graphane or fluorographene for the cases of H and F adsorption, respectively). The deposition of a reference atom to the carbon leads to the rehybridization of its bonding from sp^2^ to sp^3^, leading to a change in the chemistry of neighboring C atoms, that in turn tend to connect with other atoms. In the case of a multilayered film, such atoms are carbon atoms from the neighboring layer, which leads to the bonding of the layers to each other and the final formation of a diamond film [5].

**Figure 6:**
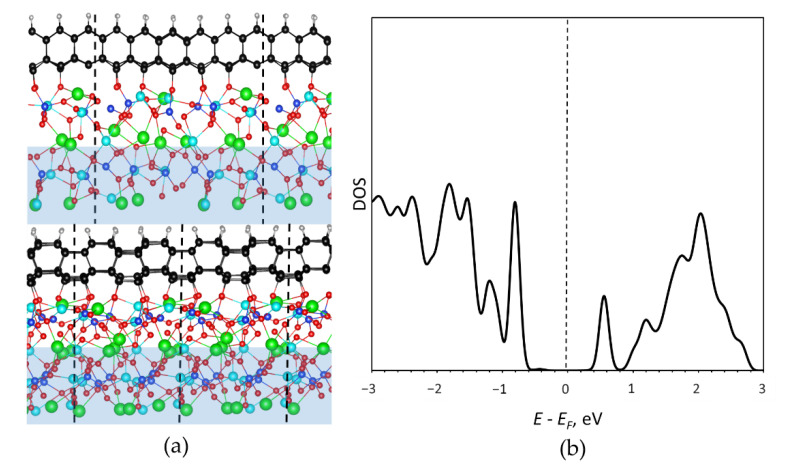
(**a**) Atomic model of a diamane film with surface $\left(10\bar{1}0\right)$ which outer side is passivated by hydrogen atoms, and the other (at the interface) is connected with oxygen atoms of the langasite substrate. Carbon, hydrogen, oxygen, lanthanum, gallium, and silicon are marked by black, gray, red, green, cyan, and blue colors, respectively. Unit cell is depicted by dashed lines. The frozen region is marked by blue; (**b**) Partial density of electronic states of the diamane (carbon and hydrogen atoms) from the diamane/langasite structure. The Fermi level is shifted to zero.

In the present case, graphene is exposed to oxygen atoms from one side and hydrogen atoms on the other side forming a Janus diamane structure. We designed the corresponding model of diamane film arranged on the LGS substrate functionalized by the hydrogen atoms from the outer side. During the relaxation surface, oxygen atoms of langasite shifted and connected with the carbon atoms on the interface-stabilizing diamane geometry. The final structure of hexagonal diamane film with the $\left(10\bar{1}0\right)$ surface (Figure 6a) showed high stability which proves the experimental suggestion of bilayer graphene diamondization by treatment by H and O- atoms. Note that the cubic diamane (111) structure was not stabilized by oxygen and was partially graphitized.

The observed high resistivity of the diamondized graphene regions is explained by the density of electronic states (Figure 6b) where formed diamane displays a band gap of ~0.7 eV (systematic underestimation by the GGA-PBE approach should be taken into account). Thus, irradiation leads to the controllable formation of regions with the large barrier in the bilayered graphene. The smaller value of the band gap (in comparison with perfect diamane which band gap is more than 3 eV [12]) can be explained by the presence of carbon atoms with unsaturated bonds at the interface not connected with oxygen.

It should be noted that the low sp^3^ carbon density observed in modified bigraphene indicates the formation of a diamane nanocluster in irradiated area. It is probably caused by the non-optimal stacking of carbon atoms in two functionalized graphene layers, which is determined by the angle of rotation between the two layers [40]. Other possible reasons are structural and technological defects that arise during the CVD growth of graphene and the process of transferring two layers of graphene onto a substrate. As per the calculations shown, not all bilayer graphene stacking can be transformed into diamane, so we can assume selectivity in the connection of twisted graphene containing areas with different packages. Therefore, fine control of stacking as well as structural and technological defects during the formation of the bigraphene/LGS structure and its irradiation with an electron beam are suggested to obtain high-quality diamane nanostructures. As a result, an experimentally observed barrier for the carriers’ transfer in the bigraphene/diamane/bigraphene nanostructure appears.

## 4. Conclusions

The presented study is devoted to the investigation of the effect of chemically induced phase transition in bilayer graphene transferred onto a langasite substrate and irradiated with a focused electron beam through a layer of polymethyl methacrylate. Transport measurements show that the resistance of the graphene bilayer after irradiation with an electron beam increases significantly, and the linear dependence of current on voltage in the bias voltage ranges from −1 to 1 V changes to a nonlinear one. This indicates the appearance of a barrier for the carriers in the irradiated region. This result is explained in the framework of the theory of a chemically induced phase transition associated with the formation of sp^3^ bonds of carbon with hydrogen and oxygen. When a certain local area of the sample is irradiated with a focused electron beam, hydrogen is released from the destroyed polymer from one side and oxygen is taken from the LGS substrate on the other side, forming stable bonds with graphene. It results in the formation of a diamane nanocluster structure in the bigraphene irradiated area. The designed model of diamane film arranged on the LGS substrate and functionalized by the H and O atoms supports the experimental observations.

## Data Availability

Not applicable.
